# Histopathological Muscle Findings May Be Essential for a Definitive Diagnosis of Suspected Sharp Trauma Associated with Ship Strikes in Stranded Cetaceans

**DOI:** 10.1371/journal.pone.0088780

**Published:** 2014-02-13

**Authors:** Eva Sierra, Antonio Fernández, Antonio Espinosa de los Monteros, Manuel Arbelo, Josué Díaz-Delgado, Marisa Andrada, Pedro Herráez

**Affiliations:** Unit of Histology and Veterinary Pathology, Institute for Animal Health, Veterinary School, University of Las Palmas de Gran Canaria, Arucas (Las Palmas), Canary Islands, Spain; Texas A&M University-Corpus Christi, United States of America

## Abstract

Ship strikes are a major issue for the conservation of may cetacean species. Certain gross and microscopic criteria have been previously reported for establishing a diagnosis of death due to ship strikes in these animals. However, some ship-strike injuries may be masked by advanced carcass decomposition and may be undetectable due to restricted access to the animals. In this report we describe histopathological muscular findings in 13 cetaceans with sharp trauma from ship strikes as the cause of death. Skeletal muscle samples were taken from the incision site and from the main locomotor muscle, the *longissimus dorsi*, in areas not directly affected by the sharp injury. The microscopic findings in tissues from both sites mainly consisted of haemorrhages; oedema; flocculent, granular or/and hyalinised segmentary degeneration; contraction band necrosis; and discoid degeneration or fragmentation of myofibres. We propose that skeletal muscle histopathology provides evidence of ante-mortem injuries even if the sample was taken elsewhere in the carcass and not only within or adjacent to the sharp trauma site and despite the advanced decomposition of some of the carcasses. This method helps to establish the diagnosis of ship strike as the cause of death.

## Introduction

Ship strikes in cetaceans are a major, worldwide conservation concern [1], [2], [3], [4], the incidence of which has increased rapidly since shipping traffic has developed on a global scale and the speed and size of the fleet has increased [5], [6]. Several measures have been proposed to protect cetacean populations from this threat, including more reliable definitive diagnoses for the reports of the number of collisions and the incidence of ship-strike mortalities [5], [6]. Ship strikes can lead to sharp trauma, in the form of severe cuts in the skin and the adjacent subcutaneous and musculoskeletal layers, as well as amputation and/or evisceration of the affected animals, which can be highly indicative of the cause of death [5], [7], [8]. However, determining the pre- or post-mortem nature of the strike is essential for the final diagnosis. Improved methodology for recognising the lesions indicative of ship strikes in cetaceans is urgently needed from the point of view of their conservation.

Several publications have established various criteria for serious injury and mortality caused by ship strikes in cetaceans and pinnipeds, which include the following: one or several cuts, proven ante-mortem bone fracture(s), and haematoma(s) and/or haemorrhage(s) [7], [8]. At the microscopic level, the typical findings of acute sharp trauma may include subcutaneous oedema and haemorrhage with myofibre degeneration, necrosis, and contracture at or beneath the site of the collision [8]. However, it is also stated that in some cases, due to the advanced state of autolysis, the restricted access to the animal, and/or peracute death before manifestation of the microscopic lesions, it is not possible to determine whether the collisions were ante- or post-mortem.

Skeletal muscle histopathological findings have been correlated with episodes of stress in a report on capture myopathy in stranded cetaceans [9], [10]. Selye [11], [12] stated that if an organism is severely damaged by an acute nonspecific nocuous agent, such as is caused by a sharp trauma, a General Adaptation Syndrome results. The severe destruction of tissue may therefore result in stress and give rise to a sequence of systemic changes that lead to lesional findings (i.e., the degeneration of cardiac and skeletal muscle) [13], [14], [15].

In this study, we designed a new muscle-based protocol for diagnosing acute sharp trauma in lethally ship-struck stranded cetaceans, which includes the histopathological findings in the muscle at the site of collision and in the *longissimus dorsi*, even if this latter muscle was not directly affected by the sharp trauma.

## Methods

### Samples

Skeletal muscle from 153 small and large odontocetes and mysticetes of 19 different species of cetaceans that were stranded on the Canary Islands were examined. The animals were of both sexes and ranged in age from neonatal to adult according to biological and morphometric parameters [16]. The species (and number examined) included fin whale (*Balaenoptera physalus*) (3), short-beaked common dolphin (*Delphinus delphis*) (10), Risso’s dolphin (*Grampus griseus*) (3), short-finned pilot whale (*Globicephala macrorhynchus*) (12), north Atlantic bottlenose whale (*Hyperoodon ampullatus*) (1), pygmy sperm whale (*Kogia breviceps*) (12), Fraser’s dolphin (*Lagenodelphis hosei*) (2), Sowerby’s beaked whale (*Mesoplodon bidens*) (1), Blainville’s beaked whale (*Mesoplodon densirostris*) (3), Gervais’ beaked whale (*Mesoplodon europaeus*) (6), harbour porpoise (*Phocoena phocoena*) (1), sperm whale (*Physeter macrocephalus*) (13), false Killer Whale (*Pseudorca crassidens*) (1), striped dolphin (*Stenella coreuleoalba*) (25), Atlantic spotted dolphin (*Stenella frontalis*) (23), spinner dolphin (*Stenella longirostris*) (2), rough-toothed dolphin (*Steno bredanensis*) (3), bottlenose dolphin (*Tursiops truncatus*) (13), and Cuvier’s beaked whale (*Ziphius cavirostris*) (19).

The skeletal muscle samples were collected during the necropsies, following standard protocols [17], over a 17-year period from 1996 through 2013. Necropsies were performed either *in situ* (on a beach or coast), or when the animals could be moved, in an alternate location suitable for necropsy. The required permission for the management of stranded cetaceans anywhere within the Canarian archipelago was issued by the environmental department of the Canary Islands’ Government. No experiments were performed on live animals because our work was based on dead stranded cetaceans, and the field studies did not involve endangered or protected species. The nutritional status of each animal was established morphologically with reference to anatomical parameters such as the presence of certain prominent bones, the dorso-axial muscular mass, and the absence or limited presence of fat, taking account of the species and the age of the animal. Using these parameters, we classified their nutritional status as good-moderate, moderate-poor, or emaciated. The carcass decomposition code was established according to Geraci and Lounsbury [16]. During the necropsies, tissues were collected from all of the major organs and the lesions and were stored in a fixative solution of 10% neutral buffered formalin for histological analysis. All of the skeletal muscle samples were taken from the middle portion of the *longissimus dorsi* muscle immediately lateral to the dorsal fin [18], [19]. The *longissimus dorsi* is part of the epaxial musculature, which lies along both sides of the vertebral column and is involved primarily in cetacean locomotion, powering the upstroke [20], [21].

Heart muscle samples were routinely collected during standardized necropsies, whenever possible, and included both left and right atrioventricular walls and valves [17], [22].

### Histochemical and Immunohistochemical Techniques

Skeletal muscle samples from all of the 153 stranded cetaceans were mounted on a tongue depressor (fixed at both ends by pins) with the myofibres oriented lengthwise and immersed in 4% neutral-buffered formalin for 24–48 hours. The same methodology was applied to all of the skeletal muscle samples, in which transversely and longitudinally oriented muscle samples were carved and routinely processed, embedded in paraffin, serially sectioned and stained with haematoxylin and eosin (HE), as well as with periodic acid-Schiff (PAS) with and without amylase digestion, phosphotungstic acid haematoxylin (PTAH), and the Von Kossa stain for specific components [23], [24], [25], [26]. In addition, the skeletal muscle samples were immunostained to detect myoglobin and fibrinogen (Dako, Glostrup, Denmark) and the fast and slow myosin heavy chain (MHC) isoforms, type II and type I, respectively, (Sigma Co., St. Louis, MO, USA), using the avidin-biotin-peroxidase method (Vector Laboratories, Burlingame, CA, USA). Paraffin-embedded sections of the skeletal muscle and kidney from a horse with exertional rhabdomyolysis were used as the positive controls for myoglobin and fibrinogen labelling. Tissue sections in which the primary antibodies were replaced by phosphate-buffered saline or non-immune serum (rabbit or mouse) were used as negative controls. A homogeneous pale red staining of myoglobin in uninjured muscles and the sarcoplasmic depletion of this protein in muscle fibres that were damaged in the ante-mortem period (demonstrable until 72 hours post mortem) is expected; whereas the intracellular accumulation of fibrinogen is exclusively observed in vital damaged muscle fibres, although immunohistochemical detection of fibrinogen is unreliable in samples with signs of advanced autolysis [27], [28]. The slow MHC antibody recognises type I fibres (slow-twitch fibres) and the fast MHC antibody recognises type IIa, IIb, and IId (IIx) fibres (fast-twitch fibres) [10], which allowed the determination of what type of fibre was most affected in each case.

### Gross and Microscopic Examination of Samples

The muscle examinations included a macroscopic evaluation of the muscle mass and a histologic examination of the muscle samples which comprised the *longissimus dorsi* muscle from all of the cases and the area surrounding the incision(s) in those animals with gross evidence of a ship strike.

All of the sections were examined in a blind manner by three veterinary pathologists and were evaluated for the following histological features: signs of degeneration/necrosis; intramyofibral inclusions/deposits and vacuoles; intramyofibral pigment deposits; chronic myopathic changes (excessive fibre size variation and increased numbers of internal nuclei); cytoarchitectural alterations (ring fibres); areas of inflammation and the presence of intramuscular parasites. The combination of the histopathological findings and the data concerning the age and the cause of death [29] was used to establish an aetiological diagnosis of muscular pathology in the majority of the animals.

All hearts taken from the stranded cetaceans were analyzed in the same manner. Gross and histolopathologic examination of heart samples included the external observation of the right and left atria and ventricles, aorta, pulmonary valve, as well as the mitral and tricuspid valves; and the microscopic study of each of the heart layers (epicardium, myocardium and endocardium) [22]. The cardiac samples from the group of the lethally ship-struck stranded cetaceans included 11 animals, due to the restricted access to the carcasses in two cases (Cases No. 2 and 3).

## Results

**Figure 1:**
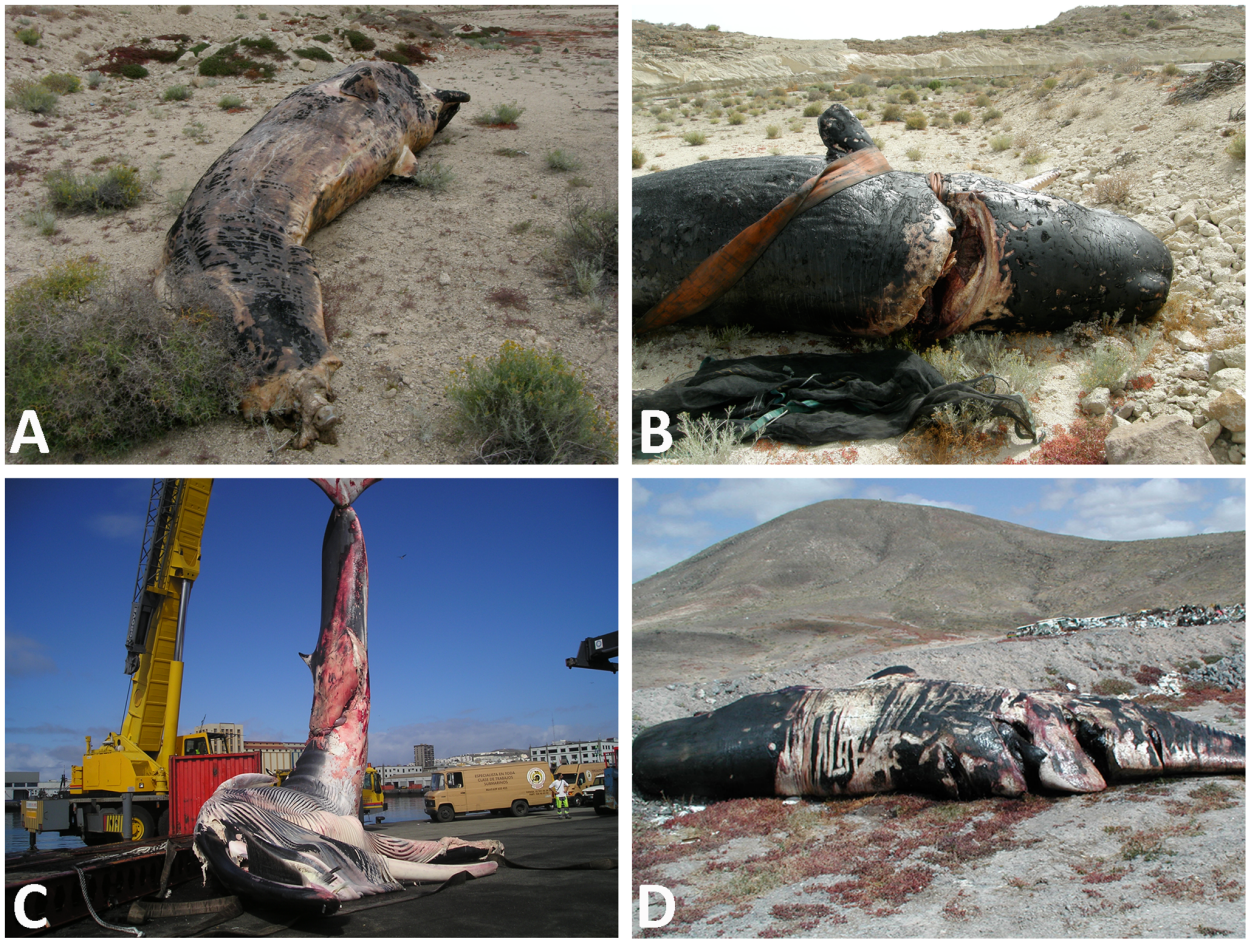
Different types of injuries from ship collisions. **A**, a male juvenile sperm whale presenting with complete amputation of the caudal fluke extending to the midlevel of the peduncle. Case No. 12; **B**, another male juvenile sperm whale presenting with a deep penetrating incision immediately caudal to the head, with skull fracture. Case No. 11; **C**, a female adult rorqual caught on the bow of a ship and carried into port. The whale presenting with a deep linear cut, with partial abdominal evisceration. Case No. 7; **D**, a female juvenile sperm whale presenting with 4 equally spaced linear cuts (i. e., propeller injury) that affected the skin, subcutaneous tissues and deep muscular tissues. Case No. 6.

Thirteen of 153 animals (8.5%) were diagnosed to have died from a ship collision (Table 1). These animals suffered severe trauma that resulted in peracute or acute death associated with a collision with a ship. Apart from one fin whale, all of the species involved in lethal ship collisions in the Canary Islands belonged to the deep-diving group. The majority of the affected animals (9/13) were sperm whales. The other 4 cases were 1 fin whale, 1 Cuvier’s beaked whale, and 2 pygmy sperm whales. All of them showed lesions indicative of severe sharp trauma. Of these animals, 3/13 showed the complete amputation of part of the body, with a linear cut; 9/13 showed a single linear cut that affected at least the skin and the subcutaneous and deep muscular tissues which in 5 cases had produced severe bone fractures and exposed cavities; and 1/13 showed 4 equally spaced linear cuts (i.e., propeller injury) affecting the skin and the subcutaneous and deep muscular tissues (Fig. 1). The carcass conditions were categorised as fresh (2/13), moderate decomposition (3/13), and advanced decomposition (8/13) (Table 1). Although the collisions were not directly observed, the types of ships involved were inferred from the type of injury, the severity of the lesion(s), and the most common types of ships operating today among the islands of the Canarian archipelago. These types of ships are mainly ferries, including one traditional monohull type and a number of different fast ferries, as high-speed crafts, and the large catamarans. In one case, the animal was caught on top of the bulbous bow of a monohull ship and transported to the port in this way. With the exception of one case involving a low-speed heavy ship with a large propeller, the lesions found were consistent with severe sharp trauma produced by large high-speed ships, with the keels producing one deadly deep cut; in some cases, the animals’ body was completely sectioned. Grossly, the skeletal muscle, particularly in the areas surrounding the incision/s, was haemorrhagic, oedematous and more friable than normal. However, the major lesions within the *longissimus dorsi* were only appreciable at the microscopic level, and all of the lethally struck animals showed microscopic musculoskeletal lesional changes of variable severity, even if this muscle had not been directly affected by the sharp trauma.

**Table 1:** Categorisation and skeletal muscle histopathological findings of cetaceans with a history of ship strikes.

Case No.	Species^a^	Estimatedage	Sex^b^	D. C.	Ship collision	Muscular lesions
**1**	*Z. c.*	Adult	F	4	C. A.	Severe multifocal extensive discoid degeneration
**2**	*P. m.*	Juvenile	F	4	I. A.	Severe diffuse hyaline and granular myonecrosis
**3**	*P. m.*	Juvenile	M	4	Very deep I. A.	Moderate diffuse discoid degeneration
**4**	*K. b.*	Juvenile	M	5	I. A.+ evisceration	Severe multifocal extensive discoid degeneration
**5**	*P. m.*	Calf	M	3	I. A.	Moderate diffuse hyaline and granular myonecrosis.Contraction-band necrosis
**6**	*P. m.*	Juvenile	F	4	P. I.	Severe diffuse hyaline myonecrosis with occasionalcontraction-band necrosis
**7**	*P. m.*	Calf	F	2	I. A.+ evisceration	Severe diffuse hyaline and granular myonecrosiswith multifocal areas of discoid degeneration.Contraction-band necrosis
**8**	*B. p.*	Adult	F	3	I. A+evisceration	Severe diffuse hyaline and granular myonecrosiswith multifocal areas of discoid degeneration
**9**	*P. m.*	Adult	F	2	I. D	Moderate multifocal hyaline myonecrosis
**10**	*P. m.*	Calf	M	3	I. A.+evisceration	Moderate multifocal hyaline and granularmyonecrosis with contraction-band necrosisand discoid degeneration
**11**	*P. m.*	Juvenile	M	4	I. A.+skull fracture	Severe multifocal extensive discoid degenerationwith contraction-band necrosis
**12**	*P. m.*	Juvenile	M	4	C. A.	Moderate multifocal hyaline myonecrosis
**13**	*K. b.*	ND	ND	4	C. A.	Severe multifocal extensive discoiddegeneration

Legend: ^a^ *B.p.*, *Balaenoptera physalus*; *K.b.*, *Kogia breviceps*; *P. m., Physeter macrocephalus, Z. c., Ziphius cavirostris.* ^b^ M, male; F, female. D. C. = Decomposition code, C. A. = complete amputation of part of the body; I. A. = incomplete amputation of part of the body by a single linear cut, P. I. = propeller injury; and N. D. = not determined.

**Figure 2:**
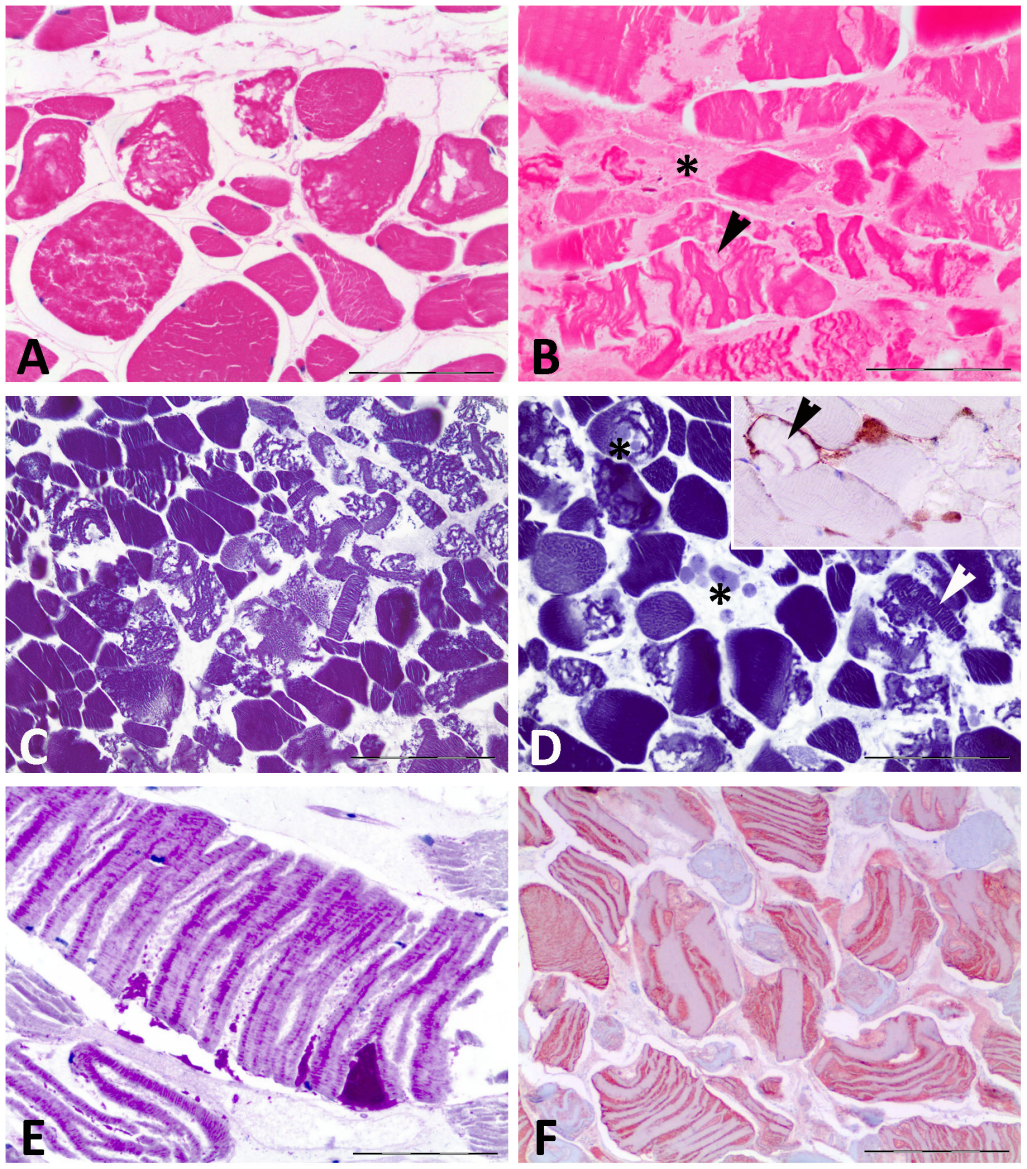
Histopathological muscle findings. **A**, cross-sectional view of a skeletal muscle sample from Case No. 8. Severe diffuse interfibrilar oedema was observed. No haemorrhages were present within the sample. Fibres varied greatly in size, many being large and swollen with flocculent, granular or/and hyalinised eosinophilic sarcoplasm. HE. Bar = 100 µm; **B**, longitudinal section of a skeletal muscle sample from Case No. 11. The degenerated myofibres with discoid degeneration displayed partial discontinuity of the sarcoplasm, which occasionally contained lysed plasma components, and an intact basal membrane (arrowhead). Fibres in advanced degeneration frequently had the appearance of nearly empty sarcolemmal sheaths (asterisk). HE. Bar = 50 µm; **C**, cross-sectional view of a skeletal muscle sample from Case No. 8. Multifocal sites of severe myofibre fragmentation that were surrounded by apparently intact fibres were present within the sample. PTAH. Bar = 100 µm; **D**, cross-sectional view of a skeletal muscle sample from Case No. 7. Multifocal sites of severe diffuse oedema were observed. Multiple sites of fragmentation and complete disorganisation of the internal structure of the affected muscle fibres were present (arrowhead). Some fibres lacking striations had formed globes within the damaged segments (asterisk). PTAH. Bar = 50 µm. Inset: Sarcoplasmic depletion of myoglobin (arrowhead) and extracellular accumulation of this protein (red globes) was appreciable in the damaged muscle fibres. Immunohistochemistry using an antibody directed against myoglobin; **E**, longitudinal section of a skeletal muscle sample from Case No. 1. Detail of the discoid degeneration of the myofibres, which in this case still retained the intramyocellular glycogen deposits. PAS. Bar = 50 µm; **F**, cross-sectional view of fibres from Case No. 1. Both types of fibres were affected by multifocal sites of severe and extensive discoid degeneration. Immunohistochemistry using an antibody directed against type II fibres. Bar = 200 µm.

The histopathological study of the skeletal muscle confirmed the haemorrhage, oedema and degeneration in the muscle fibres within the incision site, whereas the microscopic study revealed that the *longissimus dorsi* samples of the lethally struck animals showed severe acute diffuse degenerative changes or extensive multifocal degenerative changes, even though this muscle was not directly affected by the sharp injury (Table 1) (Fig. 2). The affected myofibres were characterised by the lack of cross-striations and varied greatly in size, many being large and swollen with flocculent, granular or/and hyalinised eosinophilic sarcoplasm. Multiple sites of fragmentation and complete disorganisation of the internal structure of the affected muscle fibres were observed. Despite the advanced state of autolysis of some of the carcasses in our study (8/13), these conditions were easily detected in all of the cases. When nuclei were still present in the sample (fresh to moderate decomposition code), the affected myofibres displayed pyknotic or karyorrhectic nuclei, many of which had “fallen” into the centre of the “coagulated” sarcoplasm. In five cases (38.5%), multifocal contraction-band necrosis was observed, which was identified as dense hypereosinophilic sarcoplasm running transversely across the myofibre. This feature was present in all of the calves in our study (n = 3) and in two juvenile sperm whales. Fibres in a state of advanced degeneration frequently had the appearance of nearly empty (essentially fluid-filled) sarcolemmal sheaths. Endomysial and perimysial spaces were widely expanded due to oedema and the condensation of the lysed sarcoplasmic material. Due to the acute nature of the trauma, cellular infiltrates composed of both neutrophils and macrophages were observed in only one case (Case No. 7). Both types of fibres were indistinctively affected, although fast-twitch fibres were preferentially disrupted in three of the cases (Cases No. 4, 5, and 6). In a high percentage of the animals (61.5%; n = 8), the major and more distinguishable feature was discoid degeneration or fragmentation of the myofibres. This type of myodegeneration was observed predominantly in the cetaceans in which the sharp trauma was so deep as to cause evisceration or bone fractures. This myodegenerative finding was characterised by the presence of diffuse to extensive multifocal areas of containing myofibres in which the affected segments were more closely associated than is observed in other myopathies. The degenerated myofibres displayed partial discontinuity of the sarcoplasm, which occasionally contained lysed plasma components, and an intact basal membrane. The histochemical evaluation showed the following: the affected fibres failed to show the normal dark-blue coloration PTAH staining; intramyocellular glycogen was observed in only one animal (Case No. 1) by PAS staining; and no calcification of the degenerated myofibres was reveled by Von Kossa staining in any of the cases. As expected, only those cases with a fresh decomposition code (Cases No. 7 and No. 9) showed myoglobin depletion and extracellular accumulation in the damaged muscle fibres, as well as fibrinogen immunolabelling of the injured fibres. Examination of the hearts (n = 11) showed that 10 of the 13 animals had signs of acute myocardial degeneration, mainly consisting of multifocal sites of increased acidophilic cytoplasm in the myocardiocytes, juxtanuclear vacuolisation and pyknotic nuclei. The major finding was multifocal sites of contraction-band necrosis (observed in 9 of the examined hearts; 81.8%), generally located in the subendocardial region, although subepicardial and middle region of the myocardium were also affected.

Regarding the other 140 animals with a cause of death other than a collision with a ship, discoid degeneration was not observed in any of the skeletal muscle samples from the examined specimens, including those of the same species with a similar age range (18 Cuvier’s beaked whales, 2 fin whales, 10 pygmy sperm whales, and 4 sperm whales). Acute degenerative changes with morphological features similar to those described for acute sharp ship strikes were observed in live stranded cetaceans that died very shortly after stranding (n = 16); however, the severity and tissue distribution pattern differed between these two groups, being more severe and diffuse (extending to the connective tissue) in the ship-struck animals. Further, different myofibre were affected: type II in ship-struck animals, compared to type I in live stranded animals.

## Discussion

Lethal ship-strikes detected in the Canarian archipelago involve a variety of cetacean species [5]. This region has one of the richest and most diverse populations of cetaceans in the Northeast Atlantic, with 28 different cetacean (21 odontocete and 7 mysticete) species. Although the most commonly stranded species in the area are the Atlantic spotted dolphin *Stenella frontalis* and the striped dolphin *Stenella coeruleoalba* [30], in our study, all of the species (apart from one fin whale) with trauma associated with ship collisions were characterised by performing deep and prolonged dives. It has been suggested that the prolonged period of recuperation at the surface after one or more of the episodes of apnea required by these species may be a predisposing factor [29]. Despite, the small sample size, the high proportion of calves and juveniles among the stranded ship-struck cetaceans in our study is similar to that of previously reported cases [4], [6], which supports the hypothesis that young animals may be more vulnerable to being hit by ships.

The gross lesions associated with ship collision are generally found in the areas surrounding the incision(s) and involve the skin and related soft tissues, although some bone breakage may have occurred [7], [8]. In our group of animals, gross and microscopic muscle changes within the impact site were consisted with those previously reported, which mainly consisted of haemorrhages and oedema of the relevant affected areas [7], [8]. However, the impact site is often open and therefore exposed to water and interspecific predation, which can partially or completely mask the above-described lesions.

All of the lethally struck animals displayed microscopic lesions in the *longissimus dorsi*, even when the epaxial muscles were not directly affected by the sharp injury. The microscopic findings in the muscle of this group of animals mainly consisted of oedema, acute to peracute degenerative changes (i.e., flocculent, granular or/and hyalinised eosinophilic sarcoplasm), contraction-band necrosis, discoid degeneration, fragmentation and disorganisation of the internal structure, and a minimal inflammatory reaction. These features are very similar but not identical to those described in live stranded cetaceans which were related to the stress of stranding and handling [9], [10]. The severity and acute nature of the myodegenerative processes, in combination with the extensive/diffuse pathological pattern within the muscular fascicles; and the morphological features of the affected myofibres, particularly the discoid degeneration form, which was exclusive to the animals with very deep ship-strike incisions, were common features of the stranded cetaceans with gross evidence of an acute sharp trauma associated with ship strikes in the current study, and not of the live stranded group of animals. Additionally, type I fibres have been reported to be preferentially affected by the acute degenerative changes in stress-related myopathy [10], whereas in our study, no predisposition of fibre-type injury was observed in the majority of the ship-strike cases and when present the fast-twitch type II fibres were involved. Tissue injury could be considered a stressor [11], [12], [15], [31]. Thus, the typical stress response is activated after a wounding and painful trauma [15], [32]. The first acute response to a stressor involves the activation of the hypothalamic-pituitary-adrenal axis, represented mainly by elevated adrenocorticotropic hormone (ACTH) levels [33], [34]. The peripheral effectors of this mechanism are the autonomic nervous system and the sympathomedullary circulating hormones, principally the catecholamines (epinephrine and norepinephrine). Although physiological stress has some benefits, an extreme or prolonged response with release of catecholamines and sustained, high blood cortisol and aldosterone levels are potentially damaging to the heart and the skeletal muscle [13], [35]. Contraction-band necrosis, which develops after transient ischaemia and reperfusion, is a characteristic muscle lesion associated with elevated concentrations of catecholamines [36].

In our study, contraction-band necrosis was observed in the skeletal muscle samples from 5 of the animals, which included all three of the calves in this study. Because stress may have a psychological component that can be influenced by experience, it may be expected to vary among individuals of the same species [14]. This type of lesion was very prominent in the cardiac tissue of a high proportion (n = 9) of the affected animals. This common finding in our study has also been reported in other cardiomyopathies related to psychological or traumatic stress [37]. This lesion, in addition to the perinuclear vacuolisation in myocardiocytes, is considered an indicator of acute myocardial ischaemia [35], [38]. Cardiac histopathologic changes from the lethally ship-struck stranded cetaceans were included in the current study since the coexistence of the same lesion patterns in the *longissimus dorsi* muscle and cardiac muscles suggests that they may be affected by a common mechanism [39] occurring from stressor factors [14].

Segmental myonecrosis, in the form of hyalinisation, granulation and/or flocculation of the sarcoplasm and fragmentation and disorganization of myofibres, has been described in a variety of myopathies that involved ischemic damage of the muscle [40], [41], [42]. The microscopic appearance of the muscle lesions is dependent on the interval between the time of the insult and the time of death [43]. In our study, the acute features of the lesions indicated the acute nature of the traumatic process. Focal mineralisation of affected fibres, as has been described occurring in other myopathies [44]was not observed in our study.

Discoid degeneration was observed in the skeletal muscle of the 61.5% of cetaceans (n = 8) that died by acute ship strikes. This type of necrosis was not observed in the cetaceans with myopathies associated with other etiologies in our study [10], [45], [46], [47] (unpublished data). Discoid degeneration has been described in pathologies in which hypoxia plays a major role in the production of muscle lesions [42], [48], [49], [50]. Soft tissue damage that penetrates the blubber and deeper muscle layers is often associated with the shearing and/or shredding of muscle fibres, severing of large calibre blood vessels and potential hypovolemic shock [7]. In addition, when a large calibre blood vessel is incised or transected, massive haemorrhage and exsanguination occurs, with consequent ischemic damage to drained tissues. This lesion is characterised by the fibres in adjacent sarcomeres begining to separate at the Z bands or more often by break occurring every 3–10 sarcomeres, leading to the unique and distinguishable appearance that the fibre is mummified and brittle [50], [51]. Two factors characteristic of the form of muscular degeneration have been described; the presence of I bands in stretched fibres and the amount of mitochondria in the fibres. It has been proposed that only those fibres that are stretched develop discoid degeneration; that granular degeneration develops in fibres with abundant mitochondria; and that unstretched fibres with few mitochondria acquire a homogenous hyaline appearance upon degeneration [51]. Thus, according to this proposal, fibres in rigor mortis could not be affected by discoid degeneration because hypercontracted fibres are preserved from discoid fribillar, which ignores the hypothetical situation of post-mortem insults from several ship collisions after death, when the fibres still have the capacity to contract. Vital trauma through immunohistochemically detectable depletion of myoglobin within muscle fibres and simultaneous intra-and extracellular accumulation this protein and fibrinogen in affected myofibres was possible only in animals with a fresh carcass decomposition code (n = 2), making this tool an unprofitable indicator of vital sharp trauma in this group of animals. Thus, as in our study, the majority of lethally ship-struck cetaceans showed signs of a long-post mortem interval at the time of examination, which produced extensive artefactual loss of myoglobin and the lack of immunohistochemically detectable fibrinogen [28].

In previous studies, some troubles have been described related to a conclusive diagnosis of ship strike in cetaceans and pinnipeds: advanced carcass decomposition; difficult access to the carcass; and/or peracute death. Based on our results, despite the advanced decomposition of some of the carcasses, swollen hyalinised and discoid fragmentation of myofibres were still evident in the samples, making this lesion a valuable diagnostic finding in those cases in which a definitive diagnosis of ship strike is difficult to establish. Our study also showed that histopathology of skeletal muscle provides evidence of stress/hypoxia-related injuries even if the sample is taken elsewhere in the carcass (*longissimus dorsi*) and not only within or adjacent to the site of the sharp trauma. It is well known that histopathology of wound margins and muscles can often provide insight into ante-mortem/peri-mortem vs. post-mortem injury [8], [52]. However, skeletal muscle, being one of the major organs of the body, reflects both local and systemic conditions, including stress-related trauma [10], [44]. Skeletal muscle is easily sampled during a necropsy (including in large cetaceans and when access to the cavities is impossible) and could provide essential microscopic information for a definitive diagnosis of ship strikes in cetaceans.

## Conclusions

Systematic muscle sampling should be included as a part of the necropsy protocol for stranded cetaceans, with special emphasis in the cases in which ship strikes are suspected.

The combined histopathological skeletal muscle findings described here can serve as an additional criterion for differentiating ship collision from other causes of death in these animals, when other evidence of the former is present.
